# Attributable healthcare utilization and cost of pneumonia due to drug-resistant streptococcus pneumonia: a cost analysis

**DOI:** 10.1186/2047-2994-3-16

**Published:** 2014-05-21

**Authors:** Courtney A Reynolds, Jonathan A Finkelstein, G Thomas Ray, Matthew R Moore, Susan S Huang

**Affiliations:** 1 Division of Infectious Diseases and Health Policy Research Institute, University of California Irvine School of Medicine, 100 Theory Ave, Suite 110, Irvine, CA 92697, USA; 2 Department of Population Medicine, Harvard Medical School and Harvard Pilgrim Health Care Institute, 133 Brookline Ave, 3rd Floor, Boston, MA 02215, USA; 3 Division of General Pediatrics, Boston Children’s Hospital, 300 Longwood Ave, Hunnewell G, Boston, MA 02115, USA; 4 Division of Research, Kaiser Permanente, 2000 Broadway, Oakland, CA 94612, USA; 5 Centers for Disease Control and Prevention, 1600 Clifton Road, Atlanta, GA 30333, USA

**Keywords:** *Streptococcus pneumoniae*, Antibiotic resistance, Healthcare utilization, DRSP

## Abstract

**Background:**

The burden of disease due to *S. pneumoniae* (pneumococcus), particularly pneumonia, remains high despite the widespread use of vaccines. Drug resistant strains complicate clinical treatment and may increase costs. We estimated the annual burden and incremental costs attributable to antibiotic resistance in pneumococcal pneumonia.

**Methods:**

We derived estimates of healthcare utilization and cost (in 2012 dollars) attributable to penicillin, erythromycin and fluoroquinolone resistance by taking the estimate of disease burden from a previously described decision tree model of pneumococcal pneumonia in the U.S. We analyzed model outputs assuming only the existence of susceptible strains and calculating the resulting differences in cost and utilization. We modeled the cost of resistance from delayed resolution of illness and the resulting additional health services.

**Results:**

Our model estimated that non-susceptibility to penicillin, erythromycin and fluoroquinolones directly caused 32,398 additional outpatient visits and 19,336 hospitalizations for pneumococcal pneumonia. The incremental cost of antibiotic resistance was estimated to account for 4% ($91 million) of direct medical costs and 5% ($233 million) of total costs including work and productivity loss. Most of the incremental medical cost ($82 million) was related to hospitalizations resulting from erythromycin non-susceptibility. Among patients under age 18 years, erythromycin non-susceptibility was estimated to cause 17% of hospitalizations for pneumonia and $38 million in costs, or 39% of pneumococcal pneumonia costs attributable to resistance.

**Conclusions:**

We estimate that antibiotic resistance in pneumococcal pneumonia leads to substantial healthcare utilization and cost, with more than one-third driven by macrolide resistance in children. With 5% of total pneumococcal costs directly attributable to resistance, strategies to reduce antibiotic resistance or improve antibiotic selection could lead to substantial savings.

## Background

*Streptococcus pneumoniae* (pneumococcus) causes a variety of clinical syndromes, including acute otitis media, pneumonia, and meningitis. Although several pneumococcal vaccines have been developed (including pneumococcal polysaccharide vaccine 23 (PPSV23) in 1983, pneumococcal conjugate vaccine 7 (PCV7) in 2000, and pneumococcal conjugate vaccine 13 (PCV13) in 2010), the burden of pneumococcal disease remains high across age groups, in the United States and abroad [1-10]. Our previously published model of pneumococcal burden in the United States estimated that pneumococcus was responsible for 4 million disease episodes in 2004, with acute otitis media in children accounting for the majority of cases [11]. Vaccination has led to large reductions for invasive pneumococcal diseases, yet Active Bacterial Core surveillance (ABCs) identified approximately 36,850 cases of invasive pneumococcal disease in the US in 2011 [12]. This continued high incidence of invasive pneumococcal disease is attributable to disease caused by non-vaccine serotypes [13,14].

A major concern for treatment of pneumococcal disease is the increasing frequency of antibiotic resistance [15]. These strains are primarily resistant to penicillin, macrolides and, to a lesser extent, fluoroquinolones [16]. Among the 36,850 invasive pneumococcal disease cases identified by ABCs in 2011, 9% were caused by penicillin non-susceptible strains and 26% were caused by erythromycin non-susceptible strains [12]. In addition, despite overall reductions in the incidence of resistant IPD, the prevalence of some multi-drug resistant strains, such as serotype 19A in the United States, increased due to serotype replacement and capsular switching following the introduction of PCV7 in 2000 [15,17,18]. The financial impact of antibiotic-resistant pneumococcus has not been evaluated and is likely to depend upon many factors including whether antibiotic treatment is concordant or discordant with the susceptibility profile of the infecting strain. Discordant treatment may result in delayed cure, multiple outpatient visits or hospitalization, and increases in morbidity, mortality and cost.

We used a previously developed decision tree-based model of U.S. pneumococcal disease burden to estimate the impact of antibiotic resistance upon healthcare utilization and cost [11]. We focused our analysis on pneumonia, as our prior study found that pneumococcal pneumonia accounted for 866,000 annual cases (22% of pneumococcal disease burden) and $4.9 billion dollars, or 72%, of total healthcare and time-related costs attributable to pneumococcal disease.

## Methods

We assessed the attributable burden of resistance using a previously published decision-tree model of pneumococcal disease burden in the United States in 2004 [11]. Model inputs and assumptions for an example pediatric (0- < 5 yrs) and adult (50- < 65 yrs) age group are provided in Table 1. All uses of the term “resistance” denote the combination of intermediate or high level resistance. Since minimum inhibitory concentration (MIC) breakpoints for penicillin were changed in 2008, we performed most analyses using both old and new sets of breakpoints. We assumed that the effects of antibiotic resistance occurred only in the presence of discordance between the antibiotic chosen for treatment and the susceptibility of the infecting organism. From expert opinion, we estimated what fraction of patients with discordant therapy would have delayed cure, resulting in additional outpatient visits, courses of antibiotics, or hospitalization. We further assumed that inpatient antibiotic coverage was sufficiently broad that treatment failure resulting from discordant therapy only occurred in the outpatient setting. This study was exempted from human research oversight by the Harvard Pilgrim Health Care Institutional Review Board.

**Table 1:** Inputs and assumptions for outpatient pneumococcal pneumonia for sample pediatric and adult age groups: 0- < 5 and 50- < 65 years*

				**Outcomes (% Cases)**					
				**Concordant treatment**			**Discordant treatment**		
**Sample age groups**	**Antibiotic**	**% cases receiving antibiotic**	**% cases treated discordantly**	**% simple cure**	**% delayed cure**	**% delayed cure and hospitalized**	**% simple cure**	**% delayed cure**	**% delayed cure and hospitalized**
Pediatric (0- < 5 yrs)	Amoxicillin (High dose)	9%	37%	71%	24%	5%	69%	23%	8%
	Amoxicillin (Low dose)	7%	37%	71%	24%	5%	68%	23%	9%
	Augmentin	11%	37%	71%	24%	5%	69%	23%	8%
	Azithromycin	28%	32%	71%	24%	5%	45%	15%	40%
	Oral Cephalosporin (2^nd^ generation)	7%	8%	71%	24%	5%	68%	22%	10%
	Oral Cephalosporin (3^rd^ generation)	24%	8%	71%	24%	5%	68%	22%	10%
	IM Ceftriaxone	14%	8%	71%	24%	5%	68%	23%	10%
Adult (50- < 65 yrs)	Amoxicillin	2%	37%	81%	14%	5%	77%	14%	9%
	Augmentin	19%	37%	81%	14%	5%	77%	14%	9%
	Azithromycin	15%	29%	81%	14%	5%	51%	9%	40%
	Oral Cephalosporin (3rd generation)	9%	6%	81%	14%	5%	77%	14%	10%
	IM Ceftriaxone	5%	6%	81%	14%	5%	77%	14%	10%
	Fluoroquinolones	56%	1%	81%	14%	5%	51%	9%	40%

*Derived from reference 11. Two age groups are shown as examples of model assumptions and inputs.

The incidence of outpatient pneumonia cases was obtained from the 2004–2005 National Ambulatory Medical Care Survey and 2004–2005 National Hospital Ambulatory Medical Care Survey using ICD-9 codes (all primary and secondary diagnoses) by age. We restricted outpatient cases to those visits during which an antibiotic was prescribed. Using expert panel consensus described elsewhere, we corrected for age-specific over-diagnosis among outpatient cases (16% for pneumonia) [11]. Also using expert opinion, we estimated the frequency of relevant treatment outcomes, based upon antibiotic susceptibility profiles. The distribution of agents prescribed was obtained from the National Ambulatory Medical Care Survey. The incidence of inpatient pneumonia cases was obtained from the 2004 National Hospital Discharge Survey and the 2004 National Inpatient Sample using ICD-9 codes (primary diagnosis only) by age. For cases requiring hospital admission, we estimated the likelihood of follow-up outpatient visits, nursing home stays and death from expert opinion and literature review. We used estimates of the number of inpatient and outpatient pneumonia cases in 2004 involving pneumococcal isolates resistant to penicillin, erythromycin and fluoroquinolones by age group from Active Bacterial Core Surveillance data (inpatient) and primary literature (outpatient). In our previous analyses using this model we assumed current prevalence of antibiotic resistance [11].

Cost associated with total pneumococcal disease burden (both sensitive and resistant strains) was estimated in our previously published paper using a combination of sources including the Federal Register, and public and private payor rates. We examined four categories of cost: 1) direct costs (including medical care), 2) costs from adverse outcomes, 3) work-loss costs, and 4) cost from lost wages. We conducted several sensitivity analyses related to cost in this manuscript, including an analysis for cost per hospital day. Further details on estimations of cost are provided in Appendices C-E of that manuscript [11]. To derive an estimate of healthcare utilization and costs due to medical visits and work loss (in 2012 dollars) attributable solely to resistance to penicillin, erythromycin and fluoroquinolones, we analyzed model outputs assuming only the existence of susceptible strains and calculating the resulting differences in cost and utilization. Resistant disease was assumed to be more likely to result in delayed cure, and, if not treated with concordant antibiotics, to incur additional medical and work loss costs. For outpatient cases with delayed cure, cost attributable to resistance reflected additional outpatient visits, courses of antibiotics, or hospitalization. No changes in initial antibiotic therapy were assumed.

Finally, we performed sensitivity analyses to model the projected attributable costs resulting from increases, from present levels, in resistance to penicillin, erythromycin and fluoroquinolones. We assumed antibiotic resistant strains would replace sensitive strains, resulting in stable overall prevalence of pneumococcal disease; this assumption is partly supported by several studies in children finding that overall carriage rates of pneumococcus in the nasopharynx remained stable despite serotype replacement with resistant strains after conjugate vaccination [19-21]. We further assumed that the amount of disease and frequency of treatment failure due to resistant strains would remain similar.

## Results

Overall inpatient pneumococcal pneumonia burden was estimated to be highest among those over age 65, while outpatient visits for pneumonia were most common among children less than 5 years old (Table 2). Using current MIC breakpoints, 23% (93,853 of 401,282) of inpatient cases and 34% (156,496 of 464,523) of outpatient cases exhibited resistance to at least one antibiotic. Resistance occurred most frequently to macrolides, with 20% of inpatient cases (80,932 of 401,282) and 30% of outpatient cases (139,499 of 464,523) exhibiting resistance.

**Table 2:** **Estimated pneumonia burden due to antibiotic-resistant *Streptococcus pneumoniae *** **isolates**

	**Age (years)**					
	**0- < 5**	**5- < 18**	**18- < 50**	**50- < 65**	**65+**	**Total**
**Inpatient cases**						
N (% of total)	40,386 (10%)	16,772 (4%)	42,789 (11%)	59,508 (15%)	241,827 (60%)	401,282
Susceptible isolates (pre-2008 MIC)	32,963 (82%)	14,284 (85%)	38,329 (90%)	52,804 (89%)	215,160 (89%)	353,543
Susceptible isolates (post-2008 MIC)	36,799 (91%)	15,251 (91%)	40,877 (96%)	56,559 (95%)	231,704 (96%)	381,190
PCN resistant isolates (pre-2008 MIC)*	14,375 (36%)	3,522 (21%)	8,130 (19%)	12,497 (21%)	58,038 (24%)	96,562
PCN resistant isolates (post-2008 MIC)	4,846 (12%)	1,174 (7%)	1,711 (4%)	2,975 (5%)	12,901 (5%)	23,607
ERY resistant isolates	10,821 (26%)	3,250 (19%)	7,296 (17%)	10,811 (18%)	48,754 (20%)	80,932
FQ resistant isolates	404 (1%)	168 (1%)	428 (1%)	595 (1%)	2,616 (1%)	4,211
Multiply resistant isolates (pre-2008 MIC)	8,562 (21%)	2,084 (12%)	4,927 (12%)	7,603 (13%)	36,375 (15%)	59,551
Multiply resistant isolates (post-2008 MIC)	2,926 (7%)	716 (4%)	1,095 (3%)	1,893 (3%)	8,115 (3%)	14,745
**Outpatient cases**						
N (% of total)	119,613(26%)	88,810 (19%)	116,427 (25%)	78,154 (17%)	61,519 (13%)	464,523
Susceptible isolates (pre-2008 MIC)	97,791 (82%)	72,608 (82%)	101,297 (87%)	67,216 (86%)	53,524 (87%)	392,436
Susceptible isolates (post-2008 MIC)	106,974 (89%)	79,427 (89%)	112,860 (97%)	74,729 (96%)	59,630 (97%)	433,620
PCN resistant isolates (pre-2008 MIC)	44,257 (37%)	32,860 (37%)	43,078 (37%)	28,917 (37%)	22,762 (37%)	171,873
PCN resistant isolates (post-2008 MIC)	14,354 (12%)	10,657 (12%)	9,314 (8%)	7,034 (9%)	4,922 (8%)	46,281
ERY resistant isolates	37,728 (32%)	28,012 (32%)	33,532 (29%)	22,509 (29%)	17,718 (29%)	139,499
FQ resistant isolates	1,196 (1%)	888 (1%)	1,164 (1%)	782 (1%)	681 (1%)	4,711
Multiply resistant isolates (pre-2008 MIC)	31,147 (26%)	23,126 (26%)	28,595 (25%)	19,195 (25%)	15,141 (25%)	117,204
Multiply resistant isolates (post-2008 MIC)	10,634 (9%)	7,895 (9%)	6,615 (6%)	4,968 (6%)	3,550 (6%)	33,662

*In January 2008, the Clinical and Laboratory Standards Institute revised the minimum inhibitory concentration (MIC) for non-susceptibility to penicillin.

Notes: PCN = penicillin, ERY = erythromycin, FQ = fluoroquinolone. “Resistant” includes intermediate and non-susceptible isolates. Data sources: Active Bacterial Core Surveillance data (inpatient) and primary literature (outpatient). Resistance categories are not mutually exclusive, as some strains exhibited resistance to multiple antibiotics.

To provide perspective, we also calculated resistance profiles using pre-2008 MIC breakpoints and estimated that 30% (121,503 of 401,282) of inpatient pneumococcal cases and 42% (197,680 of 464,523) of outpatient cases of pneumococcal pneumonia involved resistance to one or more of the three antibiotics studied. Using pre-2008 breakpoints, resistance would have occurred most frequently to penicillin, with nearly 50% (59,551 of 121,503) of resistant inpatient isolates and 60% (117,204 of 197,680) of resistant outpatient isolates exhibiting resistance to multiple antibiotics.

Our model estimated that resistance to penicillin, erythromycin and fluoroquinolones directly caused 32,398 excess outpatient visits and 19,336 excess hospitalizations for pneumonia (Table 3). Overall, the incremental cost of antibiotic resistance was estimated to account for 4% ($91 million) of annual pneumococcal pneumonia direct medical costs and 5% ($233 million) of total costs (including work and productivity loss). Most of the incremental medical cost ($82 of $91 million) was estimated to be due to hospitalizations resulting from erythromycin resistance. Among the 139,499 cases of outpatient pneumonia resistance to erythromycin, we estimated from expert opinion that 50,285 of these cases (36%) were actually treated with erythromycin. Among these 50,285 cases given discordant treatment, 49% still resulted in simple cure, 11% resulted in delayed outpatient resolution and 40% resulted in hospitalization due to failure to improve.

**Table 3:** Estimated annual healthcare utilization attributable to antibiotic resistant strains of pneumococcus for all ages*

	**Children (age <18)**	**Adults (age ≥18)**	**Total (all ages)**
Attributable to PCN resistance			
Hospitalizations**	867	471	1,338
Outpatient visits	1,478	780	2,258
Direct medical costs***	3,023,332	2,864,498	5,887,829
Total costs	5,358,139	8,916,750	14,274,155
Attributable to ERY resistance			
Hospitalizations	9,711	7,889	17,600
Outpatient visits	16,557	12,933	29,490
Direct medical costs	35,443,768	47,185,234	82,629,002
Total costs	68,096,627	143,766,210	211,862,838
Attributable to FQ resistance			
Hospitalizations	0	399	399
Outpatient visits	0	650	650
Direct medical costs	0	2,425,796	2,425,796
Total costs	0	6,935,208	6,935,208
Attributable to PCN, ERY, or FQ resistance			
Hospitalizations	10,578	8,758	19,336
Outpatient visits	18,035	14,363	32,398
Direct medical costs	38,467,099	52,475,527	90,942,627
Total costs	73,454,767	159,617,433	233,072,201
All cases (susceptible and resistant)			
Hospitalizations	57,158	344,124	401,282
Outpatient visits	435,967	729,334	1,165,301
Direct medical costs	265,374,908	2,295,429,821	2,560,804,729
Total costs	555,738,333	4,407,889,411	4,963,627,745

Note: PCN = penicillin, ERY = erythromycin, FQ = fluoroquinolone. “Resistant” includes intermediate and non-susceptible isolates. Post-2008 MIC breakpoints were used for penicillin resistance.

*All hospitalizations due to resistance were the result of failed therapy for patients treated initially in the outpatient setting.

**Hospitalizations include admissions from the emergency department.

***All costs are expressed in 2012 dollars. Direct medical costs exclude work and productivity loss.

While pediatric patients contributed to fewer than 15% of inpatient pneumonia cases, they constituted 45% of outpatient cases. Since costs attributable to antibiotic resistance resulted from failure of outpatient cases only, discordant treatment of pediatric cases contributed significantly to overall healthcare utilization and costs related to resistance. In particular, erythromycin resistance among patients under age 18 caused 17% of hospitalizations—that otherwise would not have occurred—and $38 million in costs.

**Figure 1:**
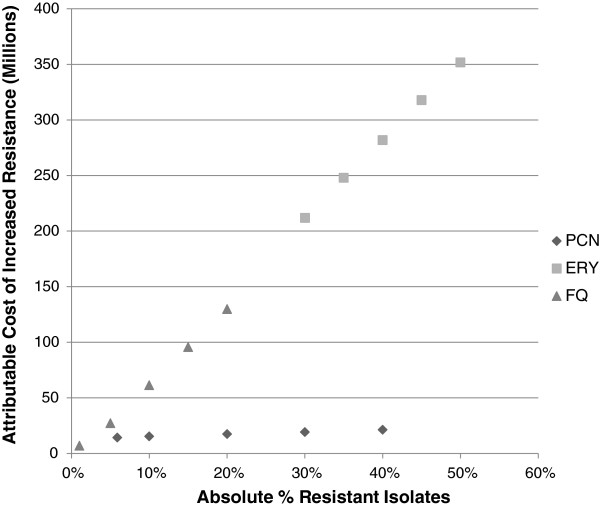
**Projected increased costs associated with increases in resistance to specific antibiotics.** Incidence of pneumococcal pneumonia is assumed to be constant. The attributable costs shown are derived from treatment failure of cases presenting as outpatients, which resulted in additional outpatient visits or courses of antibiotics, or led to hospitalizations. PCN resistance is given according to post-2008 MIC breakpoints. Cost is depicted at a given absolute percent resistance; e.g., if 20% of pneumococcal isolates were to be resistant to fluoroquinolones, the total cost attributable to that level of resistance is $127 million dollars. Initial data points for each antibiotic indicate baseline cost and resistance.

The projected costs of future increases in resistance (vs. baseline costs from 2004 estimates, expressed in 2012 dollars) to specific antibiotics are shown in Figure 1. This figure assumes a linear relationship between the number of resistant cases and the associated cost. Increased resistance to erythromycin was associated with the greatest projected cost.

## Discussion

We estimated that more than one-third of pneumococcal pneumonia cases involved resistance to penicillin, erythromycin or fluoroquinolones. While antibiotic-resistant strains accounted for a substantial portion of inpatient and outpatient pneumococcal pneumonia cases, resistance itself was responsible for a far lower percentage of total pneumococcal pneumonia medical costs (4%) and total costs (5%). This finding is due in part to the fact that discordant therapy and the resulting treatment failure were assumed to only occur in the outpatient setting. Because empiric therapy recommended by national guidelines address resistant infections, we assumed that inpatient treatment would cover resistant organisms for community-acquired pneumococcal pneumonia [22,23]. Nevertheless, costs attributable to resistance amount to over $90 million in direct medical and $230 million in total costs. Nearly all costs associated with antibiotic resistance resulted from outpatient discordant therapy that led to delays in cure, ultimately requiring additional courses of antibiotics, additional outpatient visits or hospitalization for resolution.

While resistance to penicillin was the most common, the majority of associated costs were due to erythromycin resistance. In particular, macrolide resistant pneumonia in children comprised 39% of total associated costs due to resistance, primarily resulting from hospitalization of cases initially treated in the outpatient setting. From expert opinion, we estimated that 32% of pediatric outpatient pneumococcal pneumonia cases treated with erythromycin were discordant, resulting in an additional 9700 hospitalizations annually due to delayed resolution. To address the threat of macrolide failure, recent guidelines on community-acquired pneumonia from the Pediatric Infectious Disease Society recommend amoxicillin as first line therapy, with macrolides recommended only if there is suggestion of infection with atypical organisms [22].

Based upon our projections of increased costs due to rising levels of antibiotic resistance, increases in erythromycin resistance are most likely to result in significant increases in cost. This is in agreement with our finding that delayed cure after treatment with erythromycin, predominantly in the pediatric population, is responsible for the majority of costs currently associated with antibiotic resistant pneumococcal infections. Projected increases in resistance are simply reasonable guesses based on current levels of resistance; more dramatic increases in resistance are certainly possible, and would be associated with greater increases in cost.

This study has several limitations. We estimated the impact of antibiotic resistance using an existing decision tree model of pneumococcal disease burden from 2004. The burden of antibiotic resistant cases of pneumococcal pneumonia may have changed substantially since then. We also assumed that antibiotic resistance among non-bacteremic cases was similar to bacteremic cases. In addition, the effects of the 2010 PCV13 introduction on disease burden are not reflected in these estimates. For example, emerging antibiotic resistance in replacement non-vaccine serotypes are difficult to predict and are not addressed in this pre-PCV13 model. Our model benefits from incorporation of extensive data from administrative data sources and an expert panel; however the results are highly sensitive to assumptions about the frequency of discordant therapy for which we did not directly collect data. Our model also assumed that the costs of antibiotic resistance were exclusively due to additional treatment failure for cases initially treated in the outpatient setting; this assumption does not account for the possibility of additional delayed cure among inpatients. However, we believe that the use of broad-spectrum antibiotics for inpatient treatment renders delayed cure much less likely in this setting. In our sensitivity analyses, we assumed that rising resistance would have no effect on virulence; however it has been shown that serotypes differ in their ability to cause disease and thus we likely under-estimated the projected cost associated with increased levels of antibiotic resistant strains [24]. Lastly, we assumed that further increases in resistant strains would not result in changes in empirical prescribing practices, although this is likely if levels of resistance substantially rise.

Despite the introduction of pneumococcal vaccines, the burden of pneumococcal pneumonia remains high. While we estimated that antibiotic resistance was only associated with 4% of total pneumococcal healthcare costs, the absolute cost attributed to antibiotic resistance and treatment failure was considerable. Strategies to reduce antibiotic resistance or improve antibiotic selection may prevent a significant number of hospitalizations and outpatient visits and lead to a substantial savings.

## Competing interests

The author declared that they have no competing interests.

## Authors’ contributions

JF, MM and SH determined the study design. GR built the mathematical model and ran all analyses using this model. CR composed the tables and figures and wrote the manuscript. All authors read and approved the final manuscript.
